# Deformation Properties of Thermally Aged E308L Stainless Steel During Tensile Test with Carbide Effects

**DOI:** 10.3390/ma17246070

**Published:** 2024-12-12

**Authors:** Yongming Han, Xinyuan Cao, Yonghao Lu, Tetsuo Shoji

**Affiliations:** 1 National Center for Materials Service Safety, University of Science and Technology Beijing, 30 Xueyuan Road, Beijing 100083, China; 2 Carbon Neutral Research Center, State Power Investment Corporation Research Institute (SPICRI), Beijing Future Science Park (South District), Beijing 102209, China

**Keywords:** weld overlay cladding, thermal aging, microstructure, deformation, carbides

## Abstract

Microstructure and deformation properties of both unaged and aged cladding material were studied at 400 °C for 10,000 h. The results indicated that carbide formation occurred in the cladding material, while thermal aging treatment resulted in spinodal decomposition and G-phase formation in the aged ferrite phase. Furthermore, intensive straight slip bands formed in both unaged and aged austenite phases. Continual straight slip bands formed in the unaged ferrite phase, while curvilinear slip bands formed in the aged ferrite phase during the plastic deformation process. Microcracks preferred to nucleate at the points of interaction between phase boundaries and carbides, while the aged ferrite phase experienced lowered microcrack formation along the carbide/ferrite phase boundary. Microcracks propagated along the straight slip bands in the unaged ferrite phases and curvilinear slip bands in the aged ferrite phases.

## 1. Introduction

E308L stainless steel, which is widely used as a cladding material by depositing on the surface of a low alloy steel base metal in industries with a high-temperature service environment, such as the nuclear power and petrochemical industries, possesses a duplex structure including ferrite and austenite phases and is susceptible to thermal aging embrittlement during long-term service at 320 °C [1,2,3]. It is accepted that the thermal aging embrittlement is mainly related to the microstructural variation of the aged ferrite phase [4,5,6,7], including structure decomposition and precipitation formation. According to previous works [2,7,8], spinodal decomposition and G-phase formation are considered the main microstructural changes in the ferrite phase during the thermal aging process. Spinodal decomposition induces the ferrite phase to decompose into Fe-rich and Cr-rich domains and G-phase precipitate with a chemical composition of Ni16Mn6Si7 [2,7]. G-phase material is prone to precipitate along the interdomain of the Fe-rich and Cr-rich domains [9,10]. It is widely reported that microstructural changes such as spinodal decomposition and G-phase precipitation in the aged ferrite phase result in the degradation of tensile and impact properties due to the hardness effect of the ferrite phase [4,5,7,11,12].

It is reported that long-term thermal aging significantly decreases plasticity [5,7,11], which is mainly related to the deformation mode change of the aged ferrite phase [5,13]. Li found that the hardened ferrite phase deformed by the formation of deformation twins during tensile tests, and these deformation twins might serve as susceptible sites for microcrack initiation [5]. However, Wang found that cross-slip steps occurred in the ferrite phase after thermal aging during tensile tests, and austenite/ferrite phase boundaries were the preferred sites for crack initiation [13]. These contradictory results show that the deformation behavior and crack initiation of the aged ferrite phase during in situ tensile tests need to be further studied and clarified. In addition, it is reported that carbide formation along the phase boundary might affect the microstructure and properties of stainless steels [2,12,13,14]. For the irradiated ferrite phase, these carbides induce the coarsening of G-phase precipitates in the vicinity of the phase boundary [14], and in the case of the aged ferrite phase, microcracks easily nucleate around these secondary phase particles and propagate across the hardened ferrite [13]. For cladding material, carbide formation along the phase boundary is widely reported [2,11,15], while their effects on the deformation behavior and crack initiation have drawn less attention during tensile tests. Thus, more investigation is needed.

In this study, the microstructure variation of stainless steel weld overlay cladding was studied by backscatter electron microscopy (BSE) and transmission electron microscopy (TEM). The deformation behavior of the cladding material aging at 400 °C for 0 h and 10,000 h during tensile tests was investigated by scanning electron microscopy (SEM) and BSE, and the influence of thermal aging and carbides on the deformation behavior and crack initiation of the cladding materials was discussed.

## 2. Materials and Methods

### 2.1. Materials and Thermal Aging Treatment

In order to obtain the cladding material, an E308L stainless steel weld strip with a thickness of 11 mm was deposited on A508 base metal by submerged arc strip welding (SAW). After welding, post-weld heat treatment (PWHT) at 615 °C for 16 h was used to lower the residual stress by air cooling. After PWHT, thermal aging treatment was performed at 400 °C for 10,000 h, followed by furnace cooling. As previously determined by us [16], the chemical compositions of the cladding and base metals are as shown in Table 1.

### 2.2. Microstructure Characterization

All samples for microstructure characterization were cut from the cladding materials. Samples under different conditions were gradually ground using SiC papers to 2000#, followed by polishing with a 2.5 mm diamond paste, these polished samples were observed by BSE, and further investigation on microstructural evolution was performed by TEM. For TEM observation, samples with a diameter of 3 mm were thinned by twin-jet electropolishing. Twin-jet electropolishing was carried out at 25VSCE (Olympus Corporation, Tokyo, Japan) using a solution of 10% perchloric acid and 90% methyl alcohol. FEI-F20 high-resolution transmission electron microscopy (HRTEM, Tecnai G2 F20, FEI, Portland, OR, USA), which operated at 200 KV, was carried out for TEM observation.

An MTS Nano-Indenter DCM tester with a load of 10 mN was used for the nanohardness tests. The tested samples were gradually ground using SiC papers to 2000#, followed by polishing with a 2.5 mm diamond paste. For each sample, at least three measurements were used to determine the nanohardness value of each phase.

### 2.3. Tensile Test

**Figure 1:**
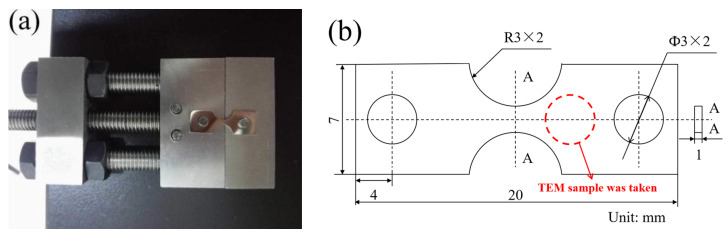
(**a**) Microscope morphology of tensile test device and (**b**) schematic diagram of tensile sample.

The in situ tensile tests were carried out in a tensile test device as depicted in Figure 1a. The schematic diagram of the tested sample is shown in Figure 1b. As shown in Figure 1a, the tested samples were strained by rotating the rod, and the rotation angle of each rotation was recorded to convert to the corresponding deformation elongation. The deformation elongation of the sample corresponded to 0.03% when the tension rod was rotated by 1°, which was calculated from 0.1 mm divided by 360°. Four deformation elongations (0%, 5.4%, 10.2%, and 12.8%) were carried out to observe the deformation behavior of the cladding material with different aging times. Before the tensile tests, all the samples were ground to 5000# grit, followed by polishing with a 2.5 mm diamond paste, and then cleaned with distilled water.

After elongation for each certain deformation, the sample was taken off the device, and the sample surface was observed by SEM and then BSE. For further investigation, TEM analysis was carried out to study the deformation mechanism of the unaged and aged cladding material using the fast Fourier transformation (FFT) and inverse FFT (IFFT) images and Digital Micrograph™ software 3.5 using HRTEM images.

## 3. Results

### 3.1. Microstructure Results

**Figure 2:**
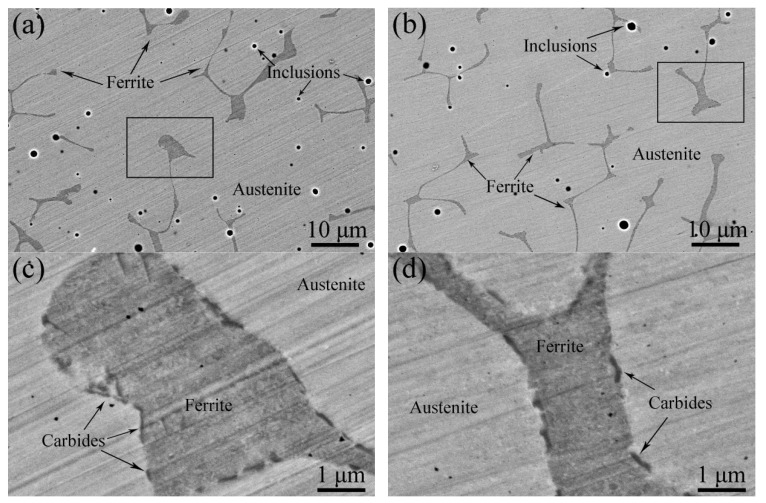
BSE images of the samples aged at 400 °C for (**a**) 0 h and (**b**) 10,000 h; (**c**) and (**d**) the corresponding magnified images of the black framed regions indicated in (**a**) and (**b**), respectively.

Figure 2a and Figure 2b show BSE images of the unaged sample and the sample aged at 400 °C for 10,000 h, respectively. It can be seen that both the unaged and aged samples have austenite phases (pale grey areas) and polygonal-shaped ferrite phases (dark grey regions), which is consistent with previous works [1,2]. In addition, there are distributed inclusions (black spots), rich in Mn, Al, Si, and O elements, which were also identified as oxides in our previous work [16]. The magnified images of the black framed regions, indicated in Figure 2a and Figure 2b, are shown in Figure 2c and Figure 2d, respectively. It is found that some carbides, with a size of not more than 1 μm, distribute around the boundary of the austenite and ferrite phases, and not much difference is found between the unaged and aged samples.

**Figure 3:**
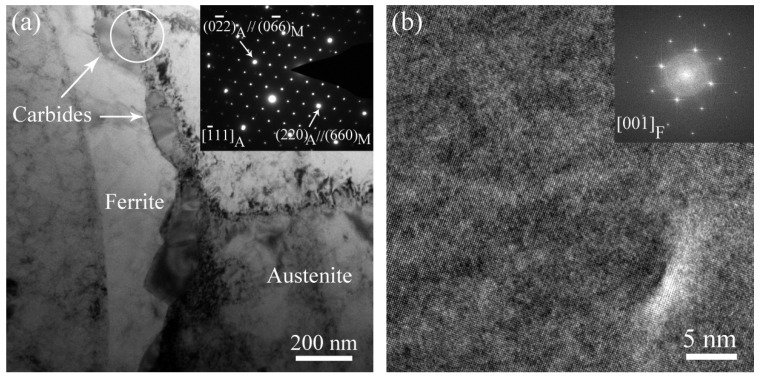
(**a**) TEM image of the unaged cladding material, the corresponding SEAD pattern of austenite and carbide is shown in the inset, and (**b**) HRTEM image of the ferrite phase, the corresponding FFT image of ferrite is shown in the inset.

Figure 3a and Figure 3b show TEM and HRTEM images of ferrite in unaged stainless steel weld overlay cladding, respectively. As shown in Figure 3a, it is obvious that nanometer-sized carbides distribute along the austenite/ferrite phase boundary, while the selected area electron diffraction (SAED) including austenite and carbides phases shown as an inset in Figure 3a reveals that these carbides exhibit a face-centered cubic (FCC) structure that has a <001>C//<001>A orientation relationship with the austenite phase. HRTEM and the fast Fourier transform (FFT) images shown in the inset in Figure 3b indicate that neither spinodal decomposition nor the other precipitation are found in the ferrite phase of the unaged cladding material.

**Figure 4:**
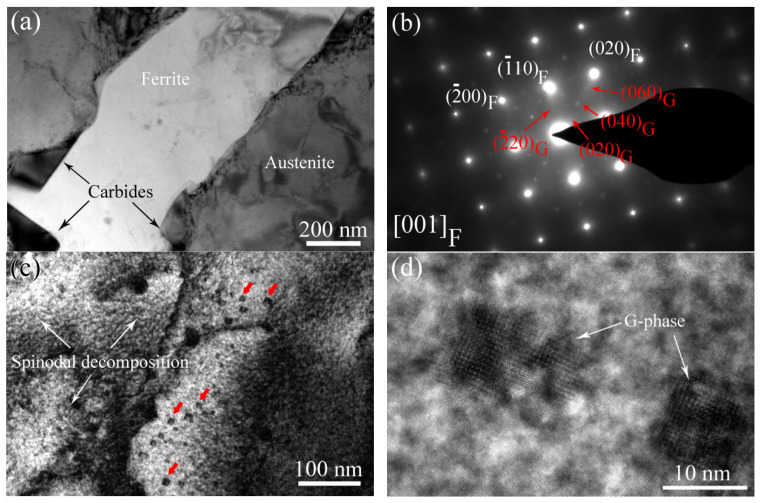
(**a**) and (**c**) TEM image of the cladding material aged at 400 °C for 10,000 h, (**b**) SEAD pattern of ferrite phase and G-phase, (**c**) and (**d**) HRTEM images of spinodal decomposition and G-phase in the aged ferrite phase, respectively.

Figure 4a shows a TEM image of the cladding material aged at 400 °C for 10,000 h. In Figure 4a, bright ferrite phase material and carbides are clearly observed along the phase boundary. In addition, the corresponding SEAD pattern in Figure 4b indicates that some precipitates form in the aged ferrite phase, showing a <001>P//<001>F orientation relationship with the aged ferrite phase. Based on previous works [9,10,17], these precipitates are G-phase precipitates. The HRTEM images of the aged ferrite phase shown in Figure 4c,d also confirm that spinodal decomposition takes place and nanometer-sized G-phases form (indicated by red arrows in Figure 4c) in the ferrite matrix.

### 3.2. Deformation Behavior of Unaged Cladding Material

**Figure 5:**
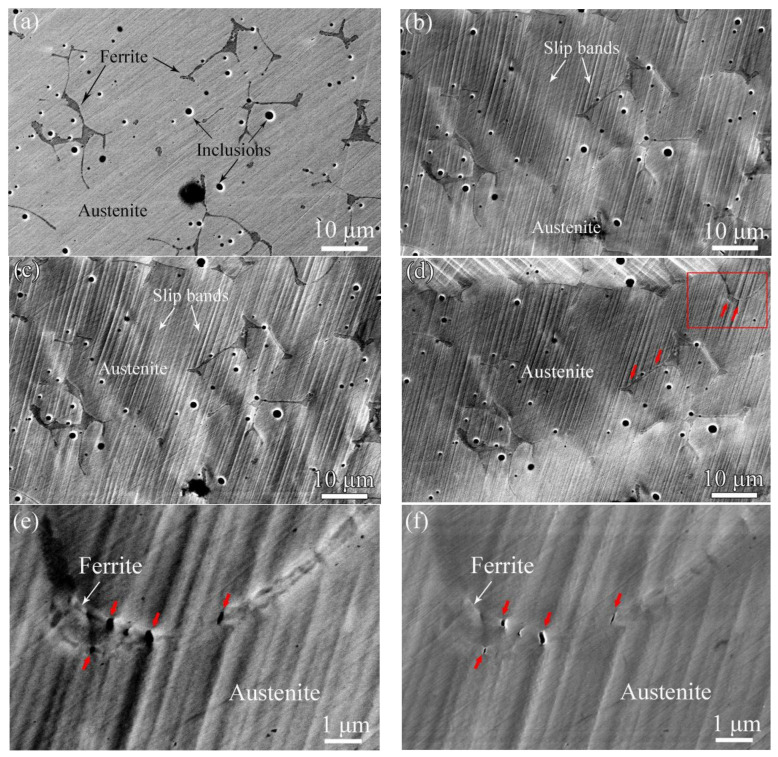
BSE images of the unaged sample with (**a**) 0%, (**b**) 5.4%, (**c**) 10.2%, and (**d**) 12.8% elongation, (**e**) and (**f**) the magnified BSE and SEM images of the red framed region indicated in (**d**), respectively.

Figure 5a–d show BSE images of the unaged sample with different deformation elongations. As shown in Figure 5a, it is found that the sample has ferrite and austenite phases. When elongation increases to 5.4%, slip bands form in the austenite phase but no obvious deformation occurs in the ferrite phase, as shown in Figure 5b. Figure 5c shows the deformation behavior of the cladding material with 10.2% elongation. It can be seen that intensive slip bands exist in the austenite matrix and there are a few slip bands across the ferrite phase, despite the existence of sharp line-shaped scratches. The deformation behavior of the unaged sample at an elongation of 12.8% is shown in Figure 5d. It can be seen that severe deformation occurs in some parts of the unaged ferrite phase, indicated by red arrows in Figure 5d. The corresponding magnified BSE and SEM images of the red framed region indicated in Figure 5d are shown in Figure 5e and Figure 5f, respectively. It is apparent that the interaction between the slip bands and austenite/ferrite phase boundary resulted in microcrack initiation across the unaged ferrite phase along the straight slip bands (indicated by red arrows).

**Figure 6:**
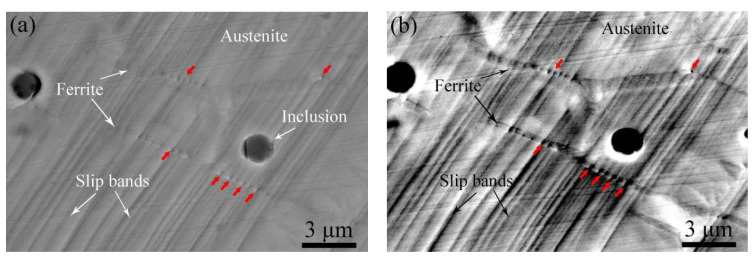

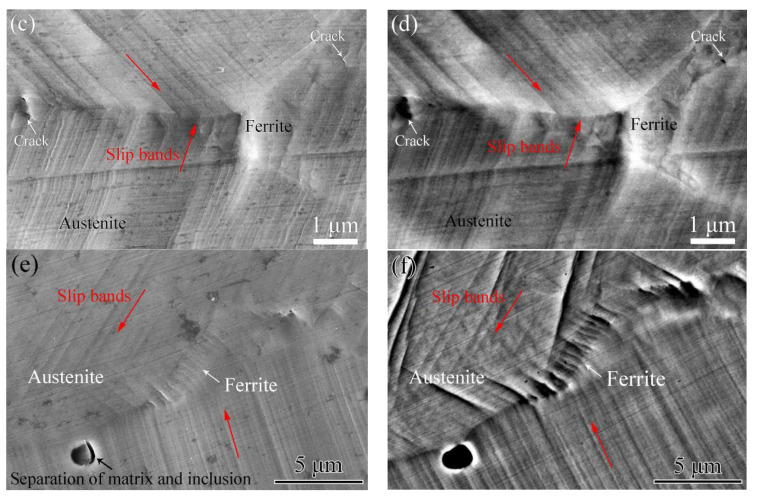
(**a**,**c**,**e**) SEM and (**b**,**d**,**f**) corresponding BSE images of the unaged sample with 12.8% elongation.

Figure 6 shows SEM and BSE images of the deformation mechanism in the unaged ferrite phase at an elongation of 12.8%. Three types of slip bands form in the unaged ferrite phase depending on the deformation behavior of the austenite phase. As shown in Figure 6a,b, the intensive slip bands, which formed in the austenite phase, cross the whole ferrite phase, and continual slip bands form in the cladding material (indicated by red arrows). For the unaged cladding material shown in Figure 6c,d, slip bands of different orientations are observed in the austenite matrix, and the slip system activated in the ferrite phase has the same orientation as one of these slip bands (indicated by red arrows). In addition, as shown in Figure 6e,f, the active slip system in the unaged ferrite phase shows a different orientation to the neighboring austenite phase, as indicated by red arrows in Figure 6e,f. In addition, separation of the matrix and inclusion is easily observed in these images. All the results indicate that the unaged ferrite phase has good plastic compatibility with the austenite matrix.

### 3.3. Deformation Behavior of Thermally Aged Cladding Materials

**Figure 7:**
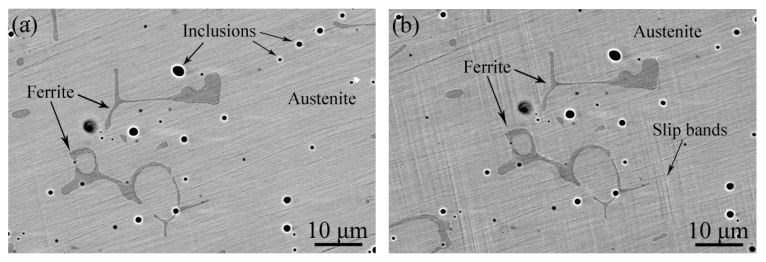

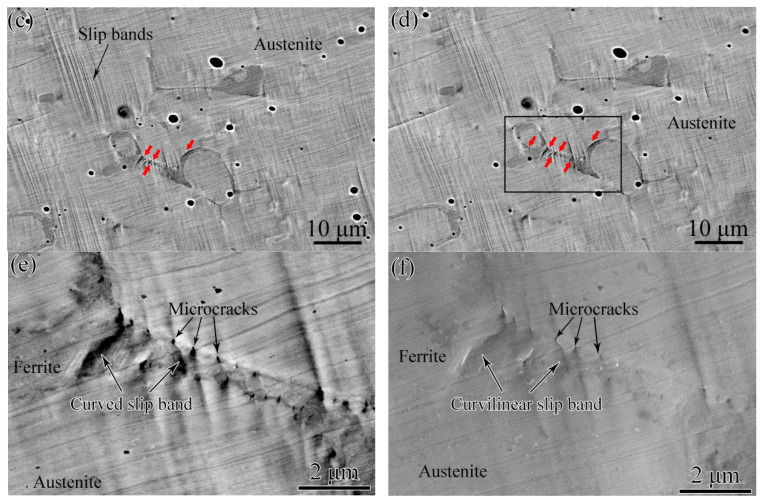
SEM image of the aged sample with (**a**) 0%, (**b**) 5.4%, (**c**) 10.2%, and (**d**) 12.8% deformation elongation, (**e**,**f**) the magnified BSE and SEM images of the black framed region indicated in (**d**).

Figure 7a–d show BSE images of the aged sample with different deformation elongations. From Figure 7a, it is found that the aged ferrite phases and inclusions are distributed across the sample. When the elongation increases to 5.4%, the activated slip system results in slip band formation in the austenite phase. For cladding material at an elongation of 10.2% as shown in Figure 6c, fine parallel slip bands form in the austenite phase that are interrupted at the phase boundary, while the ferrite phases show no obvious deformation. However, microcracks are observed in some parts of the aged ferrite phase, indicated by red arrows in Figure 7d, when the elongation increases to 12.8%. The corresponding magnified SEM and BSE images of the black framed region indicated in Figure 7d are shown in Figure 7e and Figure 7f, respectively. It can be seen that microcracks only occur in phase boundaries containing carbides, while curvilinear slip bands were observed in the aged ferrite phase.

**Figure 8:**
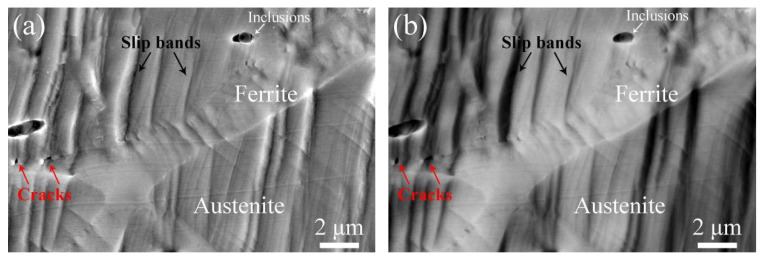

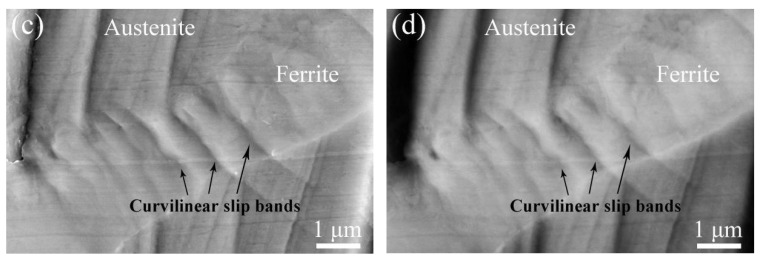
(**a**) SEM and (**c**) BSE images of the aged ferrite phase at 12.8% elongation with no carbide formation along the phase boundary, (**b**) and (**d**) the corresponding magnified images of the aged ferrite phase, respectively.

Figure 8a and Figure 8b show SEM and BSE images of the aged ferrite phase with 12.8% elongation respectively. No obvious carbides form along any of the phase boundaries. As shown in Figure 8a,b, some microcracks form along the austenite/ferrite phase boundary and it is apparent that the aged ferrite phase hardly deforms, with only a few slip bands forming. The corresponding magnified images of the ferrite phases are shown in Figure 8c,d. As shown in Figure 8c,d, it is apparent that, for the aged ferrite phase with no carbides distributed along the phase boundary, some curvilinear slip bands form in the ferrite phase after thermal aging.

**Figure 9:**
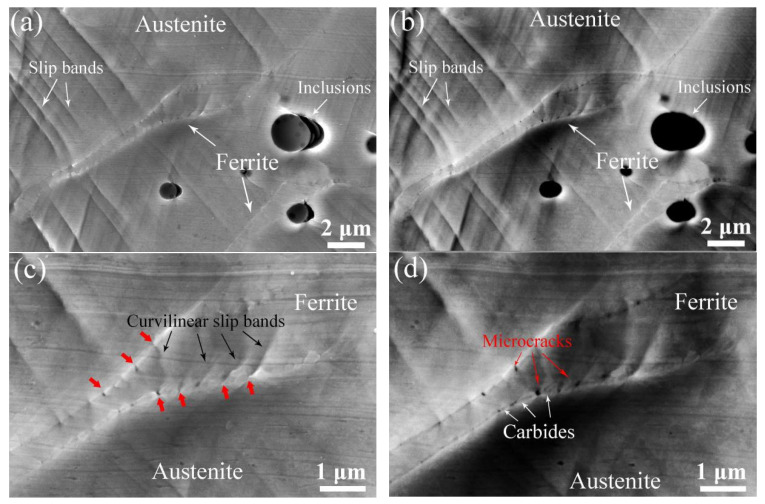
(**a**) SEM and (**b**) BSE images of the aged ferrite phase at 12.8% elongation with carbide formation along the phase boundary, (**c**) and (**d**) the corresponding magnified images of the aged ferrite phase respectively.

Figure 9a and Figure 9b show the corresponding SEM and BSE images of the aged ferrite phase with carbide formation along the phase boundary, respectively. It is found that some typical curvilinear slip bands form in the ferrite phase after thermal aging. Figure 9c and Figure 9d show the corresponding magnified images, respectively. It can be seen that these curvilinear slip bands connect to the microcracks initiated along the phase boundaries containing carbides (indicated by red arrows in Figure 9c).

**Figure 10:**
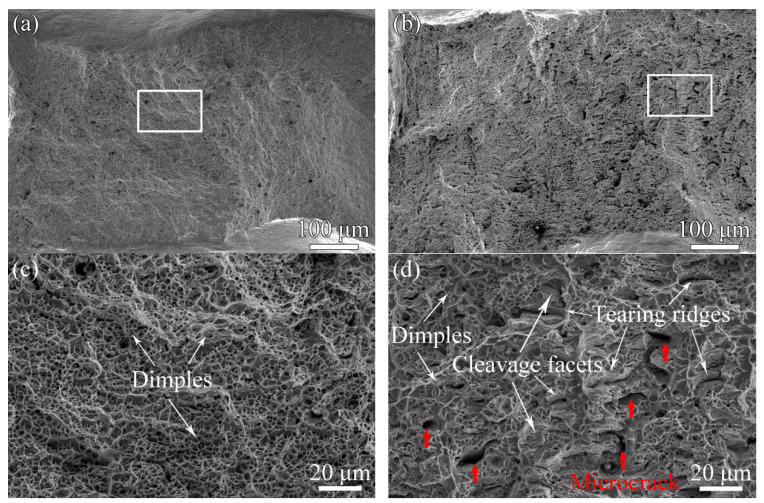
Overall appearance of the fractured samples aged at 400 °C for (**a**) 0 h and (**b**) 10,000 h, (**c**) and (**d**) the corresponding magnified images of the white framed regions indicated in (**a**) and (**b**), respectively.

Figure 10a and Figure 10b show the overall appearances of the unaged fractured sample and aged fractured sample, respectively. The unaged sample has a good plasticity and exhibits ductility features as shown in Figure 10a. The corresponding magnified image of the white-framed region indicated in Figure 10a is shown in Figure 10c. It is found that fine dimples are homogeneously distributed in the fracture surface of the unaged sample and no obvious brittle fracture features are observed. By comparison, some brittle fracture features occur in the aged sample as shown in Figure 10b and the magnified image of the white-framed region indicated in Figure 10b, is shown in Figure 10d. From Figure 10d, it is apparent that the aged sample has mixed fracture features including dimples and some brittle fracture features (tearing ridges and cleavage facets). In addition, the observed microcracks indicated by red arrows in Figure 10d confirm that the change in microstructure contributes to the embrittlement fracture of the cladding material.

### 3.4. TEM Results of the Deformed Cladding Material

**Figure 11:**
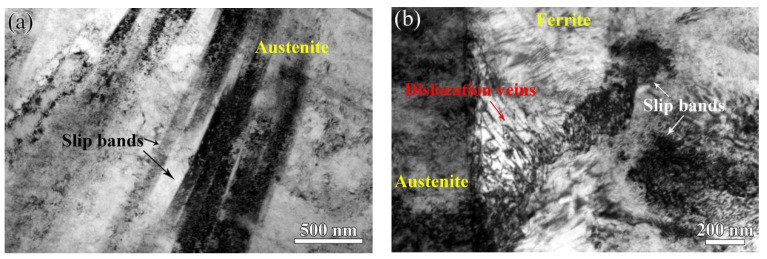

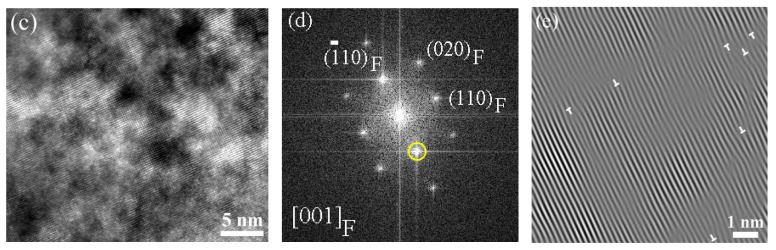
(**a**) TEM image of slip bands formed in the unaged austenite phase with 12.8% elongation, (**b**) TEM observation of unaged ferrite phase with 12.8% elongation, (**c**) HRTEM image of unaged ferrite phase, (**d**) the FFT image of (**c**), and (**e**) FFT image of (**d**).

Figure 11a shows the TEM images of the austenite phase after deformation without thermal aging. Clear slip bands form in the austenite phase. Figure 11b shows the TEM image of the unaged cladding material after deformation. It is apparent that the slip bands formed in the austenite and ferrite phases, and a dislocation vein is observed in the unaged ferrite phase. An HRTEM image of the unaged ferrite phase, as shown in Figure 11c, confirms that no obvious precipitation is found. The corresponding FFT and IFFT images are shown in Figure 11d and Figure 11e, respectively. It is apparent that some lattice distortions and dislocations existed in the unaged ferrite phase after deformation.

**Figure 12:**
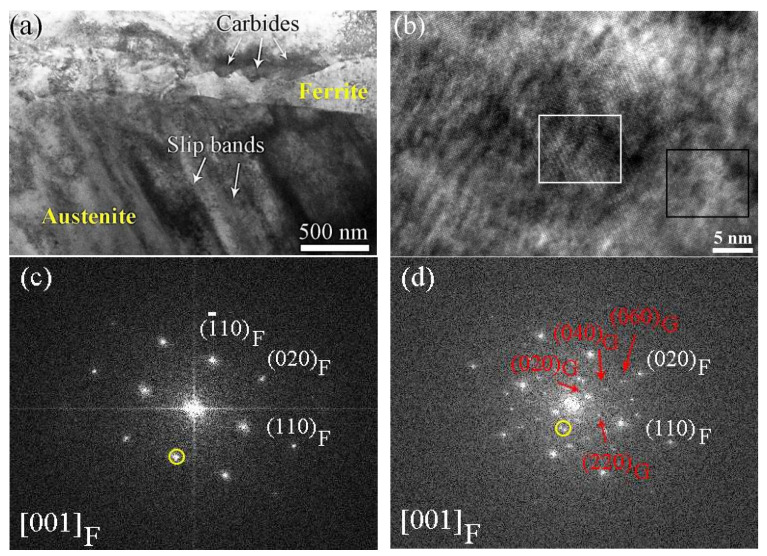

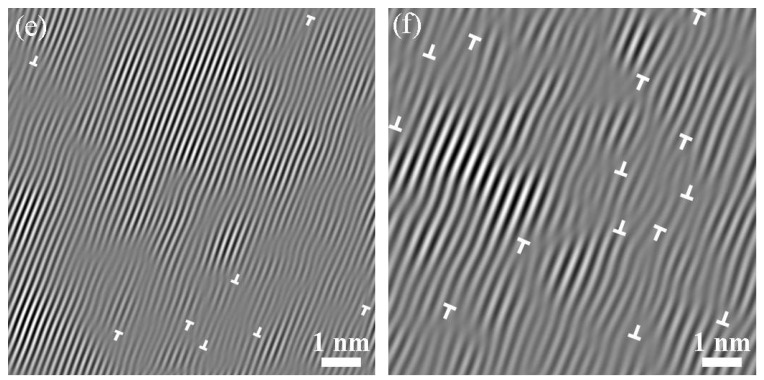
(**a**) TEM bright-field image of the aged cladding material, (**b**) HRTEM image of the aged ferrite phase, (**c**) and (**d**) the corresponding white and black framed regions indicated in (**b**), respectively, and (**e**) and (**f**) IFFT images of (**c**) and (**d**), respectively.

Figure 12a shows the bright-field TEM image of the aged cladding material after 12.8% deformation. From Figure 12a, it is found that there is no observable deformation band in the ferrite phase after aging. The corresponding HRTEM image of the ferrite phase after aging is shown in Figure 12b. It is obvious that precipitates form in the aged ferrite phase. Figure 12c,d show the FFT images of white and black framed regions indicated in Figure 12b, respectively, which indicate that the precipitate is G-phase. Figure 12e,f show the corresponding IFFT images of the white circles indicated in Figure 12c and Figure 12d, respectively, revealing that more lattice distortions exist in the G-phase and aged ferrite phase.

**Figure 13:**
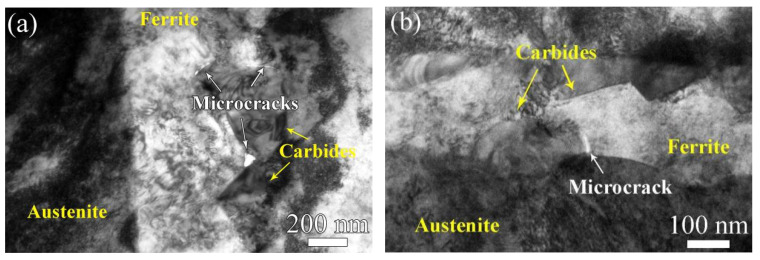
TEM images of (**a**) unaged ferrite phase and (**b**) aged ferrite phase.

Figure 13a and Figure 13b show TEM images of the unaged ferrite phase and the aged ferrite phase, respectively. From Figure 13a, it is found that microcracks with a size of about 10 nm form along the interface of the ferrite phase and carbides in the unaged sample (Figure 13a), while, for the aged cladding material, the austenite/ferrite phase boundary shows a high susceptibility for microcrack initiation, as shown in Figure 13b. All the results indicate that the hardening effect of the ferrite phase lowered the probability of microcrack formation along the carbide/ferrite phase boundary.

**Figure 14:**
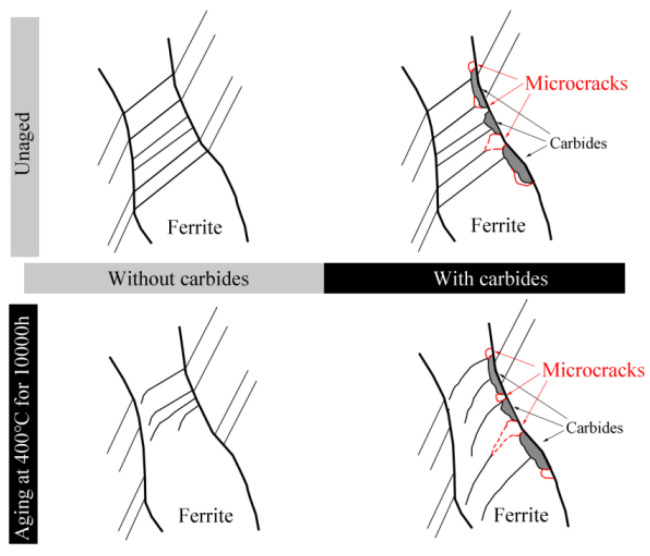
Schematic image of deformation behavior in the unaged and aged ferrite phase.

The schematic image of the deformation behavior in the unaged and aged ferrite phases is shown in Figure 14. It can be seen that a series of straight slip bands formed in the unaged ferrite and austenite phases, while carbide formation along the phase boundary had no effect on the deformation of the unaged material. For the aged cladding material, curvilinear slip bands form in the aged ferrite phase, especially for those ferrite phases with carbides formed along the phase boundary. The existence of carbides results in microcrack formation along the phase boundary, and the hardened ferrite phase lowers the probability of microcrack formation along the ferrite/carbide phase boundary. In addition, microcracks easily propagate along the straight slip bands in the unaged ferrite phase and curvilinear slip bands in the aged ferrite phase.

## 4. Discussion

### 4.1. Microstructural Evolution During Thermal Aging Process

Owing to the existence of austenite and ferrite phases in the cladding materials, a long-term thermal aging treatment could induce the occurrence of spinodal decomposition and G-phase precipitation in primarily, the ferrite phase [2,7,8,9,18,19]. In our study, the unaged cladding material showed no structural change in the ferrite phase except for the formation of some carbides along the austenite/ferrite phase boundary (Figure 3), while a mottled appearance of the aged ferrite phase, confirmed by many previous works as the typical characteristic of spinodal decomposition [7], was clearly observed (Figure 3). In addition, secondary-phase particle precipitation occurred in the aged ferrite phase. Based on previous works and our own corresponding results (Figure 12c,d), these face-centered cubic (fcc) precipitates were identified as G-phase. It is reported that G-phase elements (Ni, Mn, and Si elements) are gradually rejected by Fe-rich and Cr-rich domains and diffuse into the interdomains of Fe-rich and Cr-rich domains to form Ni-Mn-Si Clusters [7,9]. These Ni-Mn-Si clusters then serve as precursors to the G-phase, undergoing transformation into G-phase until the chemical composition meets the critical chemical composition of G-phase [7,8]. Based on the microstructural change in the aged ferrite phase during the thermal aging process, it is believed that the microstructural evolution resulted in a different deformation behavior of the ferrite phase.

### 4.2. Effect of Long-Term Thermal Aging on the Deformation Behavior

According to previous work [13,20,21], ferrite and austenite phases exhibited different deformation behaviors not only in the number of slip bands but also in the morphology of these slip bands. In our study, for the austenite phase, straight and intensive slip bands were clearly observed in the unaged and aged materials (Figs. 5 and 7), which indicated that thermal aging treatment influenced the deformation mode of the austenite phase; while the types of slip bands in the ferrite phase depended on the bulk activities of ferrite phase and the assistance deformation of the neighboring austenite phase [13,21].

According to previous work [22,23], the plastic mismatch between two materials A and B is defined as follows:

(1) $$M_{A-B}=\frac{\sigma_{A}-\sigma_{B}}{\sigma_{A}+\sigma_{B}}$$

where $M_{A-B}$ represents the degree of plastic mismatch between the two materials, and $\sigma_{A}$ and $\sigma_{B}$ are the yield strengths of material A and material B, respectively. For the phases in our study, we replace the yield strength with the hardness in Equation (1) and define the mismatch as follows:

(2) $$M_{A-B}=\frac{H_{A}-H_{B}}{H_{A}+H_{B}}$$

where $M_{A-B}$ represents the degree of plastic mismatch between the two phases, and $H_{A}$ and $H_{B}$ are the nanohardnesses of phases A and B, respectively. The obtained nanohardness results and those of previous works [24,25] and the calculated degrees of mismatch between the austenite phase, ferrite phase, and carbides are listed in Table 2.

As shown in Table 2, the hardness values of the ferrite phase are higher than those of the austenite phase in both the aged and unaged samples, which is in accordance with previous work [5], indicating that high Cr element content and thermal aging account for the hardness of the unaged and aged ferrite phases, respectively. Meanwhile, thermal aging promotes plastic mismatch and lowers plasticity compatibility between the austenite and ferrite phases. Therefore, the curvilinear slip bands, caused by independent deformation of the ferrite phase [21], formed in the aged ferrite phase (Figure 7, Figure 8 and Figure 9).

Due to the existence of a lattice parameter mismatch between the Cr-enriched and Fe-enriched domains due to spinodal decomposition, the dislocations hardly slip and then the hardening of the aged ferrite phase occurs [4,7,9,26], which explains the lower number of slip bands that formed in the aged ferrite phase (indicated by Figure 9 and Figure 10). In addition, it was reported that G-phase formation hardened the aged ferrite phase further by pinning the dislocations [27]. In our study, the existence of more lattice distortions and dislocations (Figure 12) revealed that lattice distortions increase the density of dislocations [28] and hindered the slip of dislocations in the aged materials [29,30], which then resulted in the formation of a hardened ferrite phase. Therefore, it is concluded that both spinodal decomposition and G-phase precipitation contributed to the variations in deformation behavior observed in the aged ferrite phase.

### 4.3. Effect of Carbides on the Deformation Behavior of the Ferrite Phase

Based on the results of our study, carbides showed no effect on the formation of straight and intensive slip bands in the unaged ferrite phase. Similarly, curvilinear slip bands formed in the aged ferrite phase no matter whether carbides formed. All the results indicated that carbide formation did not affect the deformation behavior of the aged ferrite phase.

However, as shown in Table 2, for the unaged sample, $M_{F-A}$ is much lower than $M_{C-A}$ and $M_{C-F}$, which indicated that microcracks initiated along the interface of the ferrite (austenite) phase and carbides as shown in Figure 13. By comparison, for the aged sample, $M_{F-A}$ showed a higher value than $M_{C-F}$, on the one hand, revealing that a hardened ferrite phase lowers the stress mismatch between the ferrite phase and carbides, and on the other hand, resulting in stress concentration at the ferrite/austenite phases boundary. Therefore, obvious curvilinear slip band formation occurred in the hardened ferrite phase (Figure 7, Figure 8 and Figure 9), and preferential microcrack initiation at the austenite/ferrite phase boundary around carbides was observed (Figure 13). Previous works also reported that carbides usually provided preferential sites for cavity nucleation during the deformation process, thereby contributing to a degradation in mechanical properties [30,31]. For the unaged cladding material, microcracks propagated along the straight slip bands in the unaged ferrite phase (Figure 5). By comparison, for the aged cladding material, the observed microcracks propagated along the curvilinear slip bands formed in the ferrite phase after thermal aging (Figure 7).

## 5. Conclusions

This study aimed to investigate the microstructure and deformation behavior of cladding materials aged at 400 °C for 0 h and 10,000 h using SEM, BSE, and TEM. The corresponding conclusions that can be drawn are as follows:

1. Carbide formation occurs in both the unaged cladding material and the cladding material aged for 10,000 h, while thermal aging treatment resulted in spinodal decomposition and G-phase precipitation in the aged ferrite phase.
2. Thermal aging showed no obvious effect on the deformation behavior of the austenite phase. Straight slip bands formed across the unaged ferrite phase, while the ferrite phase after aging deformed by the formation of curvilinear slip bands, especially for those ferrite phases with carbides formed along the phase boundary.
3. The existence of carbides resulted in microcrack formation along the phase boundary, and the presence of a hardened ferrite phase increased the occurrence of microcracks at the ferrite/austenite phase boundary. Microcracks developed along the straight slip bands in the unaged ferrite phases but along curvilinear slip bands in the ferrite phases after aging.

## Figures and Tables

**Table 1:** Chemical compositions of cladding and base metals (wt. %).

Metals	C	Cr	Ni	Si	Mn	S	P	Mo	N	Fe
E308L	0.024	20.34	10.52	0.86	1.42	0.007	0.014	<0.005	0.095	Bal
A508	0.15	0.23	0.74	0.19	1.27	0.005	0.006	0.53	/	Bal

**Table 2:** Nanohardness and degrees of plastic mismatch between the different phases. ($M_{C-A}$: the degree of plastic mismatch between carbides and the austenite phase; $M_{C-F}$: the degree of plastic mismatch between carbides and the ferrite phase; $M_{F-A}$: the degree of plastic mismatch between the ferrite and austenite phases).

	Unaged	Aging at 400 °C for 10,000 h
Austenite	2.27 GPa	2.05 GPa
Ferrite	2.7 GPa [23]	5.8 GPa [23]
Carbide	12.7 GPa [24]	12.7 GPa [24]
$M_{C-A}$	0.696	0.722
$M_{C-F}$	0.649	0.372
$M_{F-A}$	0.086	0.478
	$M_{C-A}$ > $M_{C-F}$ >> $M_{F-A}$	$M_{C-A}$ > $M_{F-A}$ > $M_{C-F}$

## Data Availability

The original contributions presented in this study are included in the article. Further inquiries can be directed to the corresponding authors.
